# Putting Cells into Context

**DOI:** 10.3389/fcell.2017.00032

**Published:** 2017-04-05

**Authors:** Sigmar Stricker, Petra Knaus, Hans-Georg Simon

**Affiliations:** ^1^Musculoskeletal Development and Regeneration Group, Institute for Chemistry and Biochemistry, Freie Universität BerlinBerlin, Germany; ^2^Cell Signaling and Regeneration Group, Institute for Chemistry and Biochemistry, Freie Universität BerlinBerlin, Germany; ^3^Department of Pediatrics, Feinberg School of Medicine, Northwestern University and Stanley Manne Children's Research InstituteChicago, IL, USA

**Keywords:** cell signaling, mechanobiology, extracellular matrix, artificial matrices, material sciences

## Cells live in a complex world

It may sound blatantly obvious, but we have to remind ourselves occasionally that *in vivo* cells experience an environment with a level of complexity far beyond experimental reach. The developing organism is a highly complex system, where each cell receives a multitude of cues of diverse nature at any given time point. Only the comprehensive integration of all these multivalent interactions determines the actual signaling state and hence the behavior of a cell.

The analysis of biological questions is mainly inspired by a reductionist approach adopted from the “exact sciences,” where it has been proven immensely successful. That is, we are used to break down our experimental setup to a manageable number of variables. This of course is inherently contradictory to the complexity of biological systems. While simplification may be the only viable option for the experimenter to dissect biological function down to detail, it has also influenced our perspective toward the experimental systems applied. For example, studies of intracellular signaling pathways are typically performed with cultured cells. Culturing cells in an *in vitro* setting became a standard model system in biomedical research and with it in cell and developmental biology. These simplified systems allow for the dissection of molecular interactions and pathways and are aimed to deepen and mechanistically understand cellular behavior. While cell cultures have generated a wealth of information into cellular function, the data obtained *in vitro* frequently are in conflict with *in vivo* observations. One reason for this discrepancy is that these analyses focus on the cell as a closed functional system, thus conceptually unhinging it from its environment.

**Figure 1:**
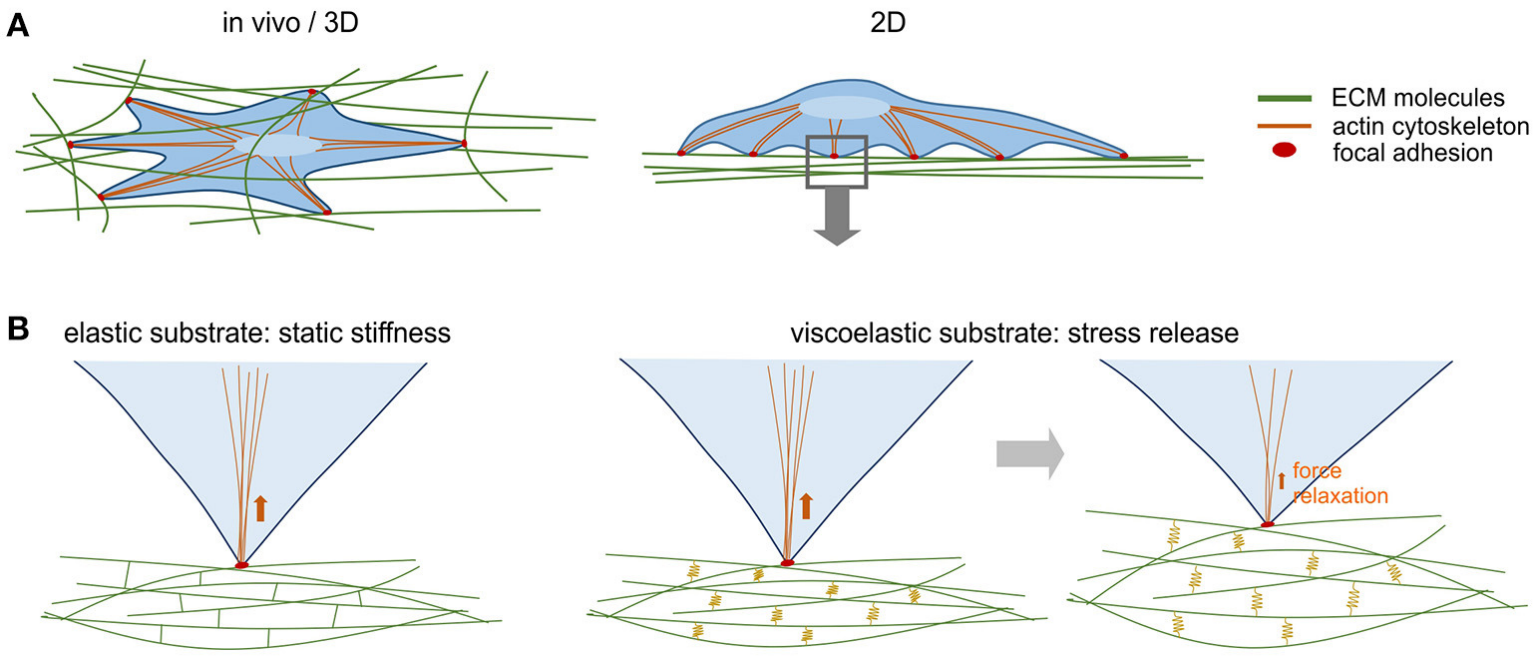
**Mechanical interactions between cells and ECM. (A)** *In vivo*, cells interact with their tissue-specific ECM mainly via focal adhesions that are linked to the actin cytoskeleton inside the cell. Via focal adhesions cells exert mechanical force to the ECM and are able to deform it and thereby measure its stiffness. In 2D *in vitro* culture, cells contact their substrate via focal adhesions on the basal side. **(B)** Standard synthetic elastic matrices where fibrillary proteins are covalently cross linked have a static stiffness profile, i.e., they deform as the cells apply force, but do not dissipate the force. The ECM in living tissues is viscoelastic and undergoes stress relaxation. Synthetic viscoelastic substrates, in which ionic crosslinking allows a certain degree of flexibility (here symbolized by springs) dissipate energy through the substrate so that the cell can gradually re-shape its ECM environment, leading to a decrease in the substrates elastic modulus over time.

In a living organism, cells are embedded in extracellular matrix (ECM) with diverse but also organ-specific properties. Cells attach to the ECM mainly via focal adhesions, which on the inside are linked to the cytoskeleton (Figure 1A). The ECM has long been seen as a mere scaffold providing support and shape; however, in the past decades it has become clear that the ECM has also an instructive character (see Adams and Watt, 1993; Tsang et al., 2010). Like a color palette ECMs come in many shades with different molecular composition, resulting in manifold chemical and physical characteristics (Rozario and DeSimone, 2010). We focus here on a rather simple but often overlooked property that provides tissues with their rigidity or elasticity. It is these mechanical properties that emerged as a decisive factor mediating information flow (see Mammoto et al., 2013). There is a multitude of interactions between cells and their ECM and it is now well accepted that cells perceive the substrate's mechanical cues and integrate them into their intracellular signal transduction pathways, gene expression and cell fate decisions. In this sense cells can be both writers and readers of ECM and its cues, implying crosstalk between cells via the ECM. Besides determining mechanical tissue properties, cells build a matrix with spatial decoration of specific growth factors to thereby modulate their local availability. Emerging data even suggest that intracellular signaling pathways integrate external biomechanical cues directly by altering the phosphorylation state of cytosolic signaling proteins (Kopf et al., 2012, 2014; Ashe, 2016).

## Biomechanical properties of the ECM and its influence on cells

For a long time it has been known that cells can, based on intrinsic sensing mechanisms, differentially respond to, for example, growth factor signaling (e.g., Nakagawa et al., 1989). The importance of the mechanical aspect, however, has only gained wider attention in recent years. In 2006, a landmark study from the Discher group showed that *in vivo* tissues exhibit an elastic modulus in the range of below 1 kPa (brain) up to over 100 kPa (ossified bone). Moreover, they provided the first evidence that this property has an instructive character on the behavior of progenitor cells, specifically mesenchymal stem cells (MSCs). The cells sensed their microenvironment by attaching and applying force to the substrate. Plating MSCs on polyacrylamide substrates of varying stiffness revealed a differentiation potential correlating with the stiffness of the *in vivo* tissue; in other words, MSCs seeded on extremely soft substrates differentiated along the neural fate, while cells seeded on hard substrates differentiated along the osteogenic fate, and intermediate stiffness substrates supported differentiation along a myogenic fate (Engler et al., 2006).

Experiments using dynamic modulation of substrate stiffness further revealed that cells initially cultured on soft (0.5 kPa) or stiff (40 kPa) polyacrylamide hydrogels and then transferred to gels of the opposite stiffness had the capacity to revert their gene expression profile from neurogenic to osteogenic, and *vice versa* (Lee et al., 2014). However, while the cells displayed a remarkable potential switching lineage specification, MSCs transferred from stiff to soft substrates maintained elevated osteogenesis markers; thus, they kept a memory of their previous culture conditions indicating a certain degree of irreversible, likely epigenetically fixed, lineage commitment.

Just recently it has been discovered that partial matrix stress relaxation is another fundamental signal in cell-ECM interactions (Figure 1B). Stress relaxation means that the force cells exert on the ECM dissipates, and over time ECM resistance decreases. Chaudhuri et al. engineered alginate polysaccharide hydrogels that are, independent from their initial elastic modulus, also tunable in their viscoelasticity. Thus, they mimic the remodeling of the matrix microenvironment over time. MSCs embedded in 17 kPa-stiff hydrogels with a rapid rate of stress relaxation demonstrated enhanced spreading, proliferation and osteogenic differentiation (Chaudhuri et al., 2016). In addition to force dissipation it is likely that remodeling of the ECM by Matrix Metalloproteinase (MMP) activity contributes to this behavior *in vivo*.

How do cells perceive these stimuli and translate them into transcriptional activity? It was shown that mechanotransduction of ECM stiffness toward MSC differentiation critically depends on YAP (yes-associated protein) and TAZ (transcriptional coactivator with PDZ-binding motif) signaling. In this context, YAP/TAZ were not activated by the “canonical” Hippo/LATS cascade, but by cytoskeletal tension and Rho-GTPase activity (Dupont et al., 2011). Interestingly, deregulation of YAP/TAZ signaling has been linked to disease conditions characterized by ECM stiffness changes such as fibrosis (Liu et al., 2015), and in this context apparently a circuit with TGFβ and WNT signaling pathways exists (Piersma et al., 2015). It is noteworthy that the interplay of ECM stiffness and mechanosensing itself impinges on the expression of profibrotic genes, driving a feed forward vicious cycle (Parker et al., 2014).

Another example are muscle resident stem cells, the so-called satellite cells (SCs). SCs reside in a specific niche underneath the myofiber's rigid basal lamina where they are kept in a quiescent state that is dependent on different factors including the collagen, glycoprotein and proteoglycan-rich ECM (e.g., Brohl et al., 2012; Bentzinger et al., 2013). In an injury situation SCs are activated and form new muscle, but importantly they also self-renew. After isolation and culture, the expansion of SCs *in vitro* and their expression of myogenic transcription factors was shown to be influenced by the elasticity of the culturing substrate (Gilbert et al., 2010), a feature recently utilized to create artificial niches maintaining satellite cell quiescence *ex vivo* (Quarta et al., 2016).

The importance of the biochemical and biophysical properties of extracellular matrices on myogenesis has been coherently demonstrated in a vertebrate *in vivo/in vitro* regeneration model. During amphibian limb and cardiac regeneration, the collagen/laminin-rich matrix typical for differentiated tissues is temporarily replaced by a transitional matrix of reduced stiffness composed of hyaluronic acid, tenascin-C and fibronectin that is surprisingly similar to the type found in developing structures (Calve et al., 2010; Mercer et al., 2013). Employing these regeneration-permissive ECMs in *in vitro* cultures, Calve et al. demonstrated an instructive role of distinct ECM components promoting cell fragmentation, proliferation, migration and differentiation of *ex vivo* skeletal muscle cells (Calve et al., 2010). In addition, using a polydimethylsiloxane (PDMS)-ECM culture system that allowed for the modulation of both, stiffness and matrix composition they could further demonstrate that ECM type and substrate stiffness over a range of 2–100 kPa combine to control migration as well as differentiation state of skeletal muscle cells (Calve and Simon, 2012).

Mechanical and biophysical properties of the ECM are provided by coordinated synthesis and secretion of matrix components with protein or sugar backbones and biologically active epitopes that result in a network of different biomolecules. Dynamic post-translational modifications including MMP cleavage further shape a characteristic local signaling environment. These native 3D structures also serve as versatile surfaces for the binding of growth factors—either in their active form or inactive preform—which, often as a consequence of mechanical stress, will be released in a spatially controlled manner. Mimicking these complex *in vivo* conditions using a 3D bioreactor with a collagen scaffold as a simplified *in vitro* culture system, the Knaus and Petersen groups provided evidence that biomechanical signaling is directly integrated into the BMP/Smad pathway (Kopf et al., 2012). Coapplication of mechanical stress and BMP stimulation resulted in increased and prolonged phosphorylation of Smads, the direct target of the transmembrane BMP receptor kinases. As a consequence, distinct target genes, including known mechanotransducers, were upregulated in a synergistic manner.

## Consequences for *in vitro* studies

Clearly, studying the complex *in vivo* interactions of cell-growth factor, cell-cell, and cell-matrix interactions and their downstream intracellular signal transduction and gene expression pathways, we will also in the future have to rely on simplified *in vitro* culture systems. As by default cells apparently integrate mechanical and biochemical inputs, the cellular behavior experimentally determined is in consequence dependent on the *in vitro* culture conditions and not necessarily reflect cellular behavior seen *in vivo*.

The standard tissue culture method is still the plastic dish, with an elastic modulus of approximately 10^6^ kPa way out of the physiological range. When a more natural environment is desired, plastic dishes are at best coated with a thin layer of mostly collagenous matrices such as gelatine or Matrigel™. This, however, rather serves as a functionalization of the surface toward better cell adherence rather than altering the mechanical properties of the substrate. In light of the growing body of evidence from the emerging field of mechanobiology we have to change course.

Time has come to move on to more comprehensive *in vitro* culture systems that better simulate the complex *in vivo* conditions. Recent approaches employing engineered biopolymers as mimetics of the natural environment provide new opportunities to develop more physiological cell culture procedures. The material sciences have made available a range of different tested hydrogels; of particular interest are those made from biologically inert polymers including polyacrylamide, PDMS, alginate, and polyethylene glycol (PEG). All of these synthetic polymers allow, to various degrees, for the tuning of stiffness over a range of 2–40 kPa (similar to that observed in natural tissues), presentation of native matrix-derived peptide epitopes, and/or binding and release of growth factors. Ideally, these cell culture models would be in a 3D architecture resembling the *in vivo* context as closely as possible. However, building a perfect mimetic of the *in vivo* environment is virtually impossible in a standard cell culture experiment when analyzing, for example, intracellular signaling using current routine reporter assays. It therefore appears as a minimal requirement for a more comprehensive experimental design to at least consider the biomechanical properties of the tissue of origin, i.e., the mechanical modulus. A realistic rational approach could be 2D culturing techniques with softer synthetic matrix substrates (as compared to the hard plastic dish) that mimic the *in vivo* viscoelastic conditions, which we think would greatly improve the reliability of *ex vivo*/*in vitro* experimentation and improve comparability to *in vivo* data. It is important to note that the fabrication of such biomimetic matrices in the laboratory is still challenging and coupled to an operating expense that clearly hinders their standard application. However, custom products are beginning to enter the market and it is foreseeable that a panel of matrix solutions will become available in the near future tailored to many if not most individual experimental needs.

Importantly, embracing more physiological cell culture conditions might generate a fundamentally new understanding of how extracellular cues, both insoluble and soluble, are integrated and stored to guide cellular behavior. These immediate biological goals would further help to achieve current and future therapeutic challenges in humans (see Sommerfeld and Elisseeff, 2016).

## Author contributions

All authors listed, have made substantial, direct and intellectual contribution to the work, and approved it for publication.

### Conflict of interest statement

The authors declare that the research was conducted in the absence of any commercial or financial relationships that could be construed as a potential conflict of interest.
